# Advances in the differential molecular diagnosis of vesicular disease pathogens in swine

**DOI:** 10.3389/fmicb.2022.1019876

**Published:** 2022-10-25

**Authors:** Wenxian Chen, Weijun Wang, Xinyan Wang, Zhaoyao Li, Keke Wu, Xiaowen Li, Yuwan Li, Lin Yi, Mingqiu Zhao, Hongxing Ding, Shuangqi Fan, Jinding Chen

**Affiliations:** ^1^College of Veterinary Medicine, South China Agricultural University, Guangzhou, China; ^2^Guangdong Laboratory for Lingnan Modern Agriculture, Guangzhou, China; ^3^Key Laboratory of Zoonosis Prevention and Control of Guangdong Province, Guangzhou, China

**Keywords:** FMDV, SVA, SVDV, PCR, isothermal amplification, Luminex, CRISPR-Cas, differential diagnosis

## Abstract

Foot-and-mouth disease virus (FMDV), Senecavirus A (SVA) and swine vesicular disease virus (SVDV) are members of the family Picornaviridae, which can cause similar symptoms - vesicular lesions in the tissues of the mouth, nose, feet, skin and mucous membrane of animals. Rapid and accurate diagnosis of these viruses allows for control measures to prevent the spread of these diseases. Reverse transcription-polymerase chain reaction (RT-PCR) and real-time RT-PCR are traditional and reliable methods for pathogen detection, while their amplification reaction requires a thermocycler. Isothermal amplification methods including loop-mediated isothermal amplification and recombinase polymerase amplification developed in recent years are simple, rapid and do not require specialized equipment, allowing for point of care diagnostics. Luminex technology allows for simultaneous detection of multiple pathogens. CRISPR-Cas diagnostic systems also emerging nucleic acid detection technologies which are very sensitivity and specificity. In this paper, various nucleic acid detection methods aimed at vesicular disease pathogens in swine (including FMDV, SVA and SVDV) are summarized.

## Introduction

Picornaviruses are small non-enveloped viruses containing a single-stranded and positive-sense RNA protected by an icosahedral capsid (Holland et al., 1960; Kim et al., 2016). Picornaviruses seriously affect the respiratory system, digestive system and central nervous system, and cause a series of inflammation and lesions in organs and tissues such as heart, liver and skin (Zell, 2018). Picornaviruses are omnipresent and distributed worldwide. Studies have shown that the number of newly identified picornaviruses has increased dramatically over the past decade, and they become important pathogens that endanger human and animal health (Palmenberg et al., 2009; Lewis-Rogers and Crandall, 2010; Tapparel et al., 2013; Zell, 2018). Foot-and-mouth disease virus (FMDV), Senecavirus A (SVA) and swine vesicular disease virus (SVDV) are members of the family *Picornaviridae*, which can cause similar symptoms - vesicular lesions in the tissues of the mouth, nose, feet, skin and mucous membrane of animals. All three of them can threaten the development of animal industry and national economy.

FMDV, classified into the genus *Aphthovirus* in the family *Picornaviridae*, is still one of the important animal disease pathogens of economic concern (Rodriguez-Habibe et al., 2020; Brown et al., 2021). Based on genetic and antigenic analyzes, FMDVs throughout the world have been geographically divided into seven serotypes (O, A, Asia1, C, SAT1, SAT2, SAT3). FMDV has a broad host range to all cloven-hoofed animals including domestic and wild ruminants and pigs. (Grubman and Baxt, 2004; Rodriguez-Habibe et al., 2020). Animals infected with FMDV often present with symptoms of fever and blisters on the mouth and hooves, with low mortality but high morbidity, leading to production losses. FMDV can contaminate the environment through aerosols and cause long-distance transmission events, so that it complicates the control of Foot-and-mouth disease (FMD) outbreaks (Gloster et al., 2003; Brown et al., 2022).

SVA is the only member of the genus *Senecavirus* in the family *Picornaviridae*, which was discovered in 2002 and once named Seneca Valley virus 001 (SVV-001; Hales et al., 2008). Initially, it was used in different human cancer treatment research as its potent oncolytic activity (Reddy et al., 2007; Burke, 2016). Subsequently, pigs with idiopathic vesicular disease (IVD) like clinical symptom occurred in Canada (Pasma et al., 2008), America (Singh et al., 2012) and Brazil (Leme et al., 2015; Vannucci et al., 2015) were confirmed to be infected with SVA. In 2015, the pathogen of vesicular lesions occurred in a pig farm in Guangdong Province of China was identified as SVA (Wu Q. et al., 2017), and then SVA gradually spread to other provinces in the following years (Liu et al., 2020).

SVDV, the causative agent of Swine vesicular disease (SVD), was first diagnosed in Italy in 1966 (Nardelli et al., 1968). SVDV belongs to the *Enterovirus* genus in the family *Picornaviridae* and was considered to be a porcine variant of human coxsackievirus B5 due to their close serological relationship (Graves, 1973). The disease only affects pigs and does not have a serious impact on production. However, SVD has similar clinical symptoms to FMD, which can pose a threat to international trade (Lin and Kitching, 2000; Dibaba, 2019).

These pathogens are difficult to diagnose clinically due to the similar vesicular lesions observed on infected animals. With the development of biotechnology, molecular biology diagnostic technology is constantly innovating and plays an important role in the diagnosis and differentiation of different viruses. In this paper, we mainly describe available and novel emerging diagnostic methods and multiplex assays for the detection of FMDV, SVA and SVDV. Tables 1–3 summarizes some recent studies on molecular diagnostic assays for the detection of FMDV, SVA and SVDV, respectively.

**Table 1:** Molecular diagnostic assays for detection of FMDV infection.

Detection method	Detection serotype	Detection target	Amplification temperature and time	Display of result	Limit of detection	References
RT-PCR	O/A/C/Asia 1 universal	5’UTR	-	Agarose gel electrophoresis	10^0^ to 10^−2^ dilution	Reid et al. (2000)
One-step multiplex RT-PCR	Detection and differentiation of Vietnamese FMDV serotypes O, A, and Asia 1 directly	2B and VP1	-	Agarose gel electrophoresis	O: 10^2.5^ TCID_50_/mL Asia1: 10^3.5^ TCID_50_/mL A: 10^1.5^ TCID_50_/mL	Le et al. (2011)
RT-PCR	7 serotypes are universal	L and P1	-	Agarose gel electrophoresis	10^−1^ to 10^−3^ dilution	Xu et al. (2013)
RT-PCR	7 serotypes are universal	3D	-	Agarose gel electrophoresis	10^0.2^ to 10^−2.8^ TCID_50_/mL	Nishi et al. (2019)
Real-time RT-PCR	7 serotypes are universal	3D	-	Fluorescent intensity in real-time thermal cycler	10 and 100 virus genomes/vol tested	Callahan et al. (2002)
Real-time RT-PCR	detection and differentiation of serotype O, A and Asia-1 in the Middle East	VP1	-	Fluorescent intensity in real-time PCR Thermocycler	1.78 to 2.74 copies of in vitro–transcribed FMDV RNA depending on serotypes	Reid et al. (2014)
Real-time RT-PCR	all 7 serotypes	3D and 5’UTR	-	Fluorescent intensity in real-time PCR Thermocycler	The detection limit of RT-qPCR (with tailed primers) targeting 3D and 5′ UTR of FMDV are −0.72 and −0.35 log10 TCID50/mL of FMDV O1 Manisa, respectively	Vandenbussche et al. (2017)
A fully automated cartridge-based real-time RT-PCR diagnostic system	All 7 serotypes	3D	-	Fluorescent intensity in real-time PCR Thermocycler	10^−5^ to 10^−6^ dilution of the FMDV O/UAE 2/2003 stock depending on the nuclei acid extraction kits	Goller et al. (2018)
RRT-PCR	Six serotypes (O, A, Asia 1, SAT 1, 2 and 3)	3D	-	Fluorescent intensity in handheld Biomeme two3™ Real-Time PCR Thermocycler (two3)	10^−4^, 10^−3^, 10^−2^, 10^−5^, 10^−3^, and 10^−3^ dilutions of FMDV O, A, Asia 1, SAT 1, SAT 2, and SAT 3 stocks, respectively	Hole and Nfon (2019)
Multiplex real-time RT-PCR	Detection and serotyping of serotype O, A and Asia-1	VP1	-	Fluorescent intensity in real-time PCR detection system	O: 10^1^ TCID_50_/mL A: 10^1^ TCID_50_/mL Asia1: 10^2^ TCID_50_/mL	Lim et al. (2022)
RT-iiPCR	All 7 serotypes	3D	-	Utilizes a commercially available compact, portable POCKIT™ Nuclei Acid Analyzer (GeneReach, USA) for rapid (<2 h) detection	≥9 copies of in vitro–transcribed FMDV O1 Manisa/69 3D RNA	Ambagala et al. (2017)
RT-ddPCR	serotypes O, A, and C	3D	-	PCR amplification of cDNA target in the droplets, the plate containing the droplets was placed in a QX200 droplet reader	10^1.4^ TCID_50_/mL and 26.5 copies of viral RNA	Pinheiro-de-Oliveira et al. (2018)
RT-LAMP	all 7 serotypes	2B	Be performed at 64°C for 45 min and then terminated by heating at 80°C for 10 min.	Agarose gel electrophoresis	10 copies of FMDV RNA per reaction	Chen et al. (2011)
RT-LAMP	Serotyping of serotype O, A and Asia1	P1	Incubation at 63°C for 60 min	Agarose gel electrophoresis	O: 10^−3^ TCID_50_ A: 10^−5^ TCID_50_ Asia1: 10^−3^ TCID_50_	Madhanmohan et al. (2013)
RT-LAMP	Serotype O	VP3	Incubation at 62°C for 40 min and then terminated by heating at 80°C for 5 min	By visual detection of a color change from purple to sky blue due to the presence of the metal ion indicator, HNB.	10^2^ TCID_50_/mL and 10^3^ copies/μL	Lim D. et al. (2018)
RT-LAMP	Serotype A	VP1	Be completed in 40 min at 62°C	By visual detection of a color change from purple to sky blue due to the presence of the metal ion indicator, HNB.	10^2^ TCID_50_/mL	Lim D. R. et al. (2018)
RRT-LAMP	All 7 serotypes	3D	Positive assay signals were generated within 15 min for the lowest concentration of a standard RNA sample at 62°C	Real-time fluorescence values of 6-carboxyfluorescein (FAM)-labeled probe were measured in on-going reactions at the end of each annealing step.	10^2^ copies/μL	Lim et al. (2020)
RT-RPA	All 7 serotypes	3D	Incubation at 42°C for 20 min.	Fluorescence detection in the FAM channel (excitation 470 nm and detection 520 nm) was performed in an ESEQuant tubescanner (Qiagen Lake Constance GmbH, Stockach, Germany) at 42°C for 20 min	10^2^ RNA molecules	Abd et al. (2013)
RPA-LFD	All 7 serotypes	2B	Incubation at 38°C within 20 min	Be visualized by LFD duplexes labeled with anti-FAM gold conjugates and anti-Biotin antibodies (Milenia Biotec GmbH, Germany)	10 copies of plasmid	Wang H. M. et al. (2018)
RPA-LFD	Differentiate FMDV serotypes A, O or Asia 1, respectively	VP1	Incubation at 38°C for 20 min	Be visualized by LFD	3 copies of plasmid DNA or 50 copies of viral RNA per reaction	Wang H. et al. (2018)
LFS RT-RPA	Serotypes O, A and Asia1	3D	Be performed in a closed fist using body heat for 15 min, and the products were visible on the LFS inspected by the naked eyes within 2 min	Be performed in a closed fist using body heat for 15 min, and the products were visible on the LFS inspected by the naked eyes within 2 min	10^2^ copies	Liu et al. (2018)

**Table 2:** Molecular diagnostic assays for detection of SVA infection.

Detection method	Detection target	Amplification temperature and time	Display of result	Limit of detection	References
RT-PCR	VP3/VP1 region	-	Agarose gel electrophoresis	-	Leme et al. (2015)
nest-PCR	VP1	-	Agarose gel electrophoresis	0.0181 ng/μL of cell-cultured SVA isolate	Feronato et al. (2018)
rRT-PCR	3D	-	Fluorescent intensity in real-time PCR detection system	0.79 TCID_50_/mL	Fowler et al. (2017)
TaqMan-based qRT-PCR	VP1	-	Fluorescent intensity in real-time PCR detection system	1.3 × 10^1^ genomic copies/μL	Dall et al. (2017)
Real-time RT-PCR	5’ UTR	-	Amplification reactions were performed on an ABI 7500 Fast instrument (Thermo Fisher Scientific).	About 3.5 RNA copies per reaction	Zhang J. et al. (2019)
RT-ddPCR one-step	3D	-	The QX200 droplet reader (BIO-RAD, USA) was used to analyze each droplet individually, which counts positive and negative droplets to establish absolute quantification of samples (concentration).	0.185 TCID_50_ of virus and 0.1 fg of SVA plasmid	Pinheiro-de-Oliveira et al. (2019)
RT-ddPCR	3D	-	After amplification, the droplets from each well of the plate were read individually by a QX200™ Droplet Reader (Bio-Rad), and the data were analyzed with QuantaSoft™ software (Bio-Rad)	1.53 ± 0.22 copies of SVA RNA	Zhang Z. et al. (2019)
RT-iiPCR	3D	-	Be placed into a POCKIT™ Nucleic Acid Analyzer for RT-PCR reaction	7 RNA copies per reaction	Zhang J. et al. (2019)
Real-time RT-LAMP	VP2	Incubation at 63 °C for 1 h.	Be detected a Thermostatic Fluorescence Detector DEAOU-308C	1 TCID_50_/mL	Zeng et al. (2018)
RT-LAMP LFD	3D	Incubation at 61^o^C for 50 min.	Be visualized by LFD	4.5 × 10^−8^ ng/μL	Li et al. (2019)
rRT-RAA	VP2	Incubation at 42^o^C for 30 min	Fluorescence signal was automatically recorded in real-time by CFX96 Bio-Rad real-time PCR instrument.	1.185 TCID_50_	Wang et al. (2021)
RPA-LF	VP1	Incubation at 35°C for 25 min	Be visualized by LFD	15 copies/μL	Wang et al. (2022)

**Table 3:** Molecular diagnostic assays for detection of SVDV infection.

Detection method	Detection target	Amplification temperature and time	Display of result	Limit of detection	References
RT-PCR	Parts regions of the structural proteins 1C and 1D	-	Agarose gel electrophoresis	100 TCID_50_	Lin et al. (1997)
RT-nPCR	Parts regions of the structural proteins 1C and 2A	-	Agarose gel electrophoresis	0.1 TCID_50_	Lin et al. (1997)
Double PCR	The simultaneous Detection of SVDV and ASFV P72	-	Agarose gel electrophoresis	SVDV: 7.6 × 10^2^ copies/μL ASFV: 1.5 × 10^5^ copies/μL	Peng et al. (2015)
RT-PCR	3D	-	Agarose gel electrophoresis	1 TCID_50_	Pezzoni et al. (2020)
Real-time one-step RT-PCR	2C	-	Fluorescent intensity in real-time PCR detection system	2 × 10^2^ copies/μL	McMenamy et al. (2011)
Real-time RT-PCR	5’ UTR	-	Fluorescent intensity in real-time PCR detection system	10^−6^–10^−7^ dilution of UKG 27/72 SVDV isolates	Reid et al. (2004b)
rtRT-PCR SYBR Green	3D	-	Fluorescent intensity in real-time PCR detection system	10 TCID_50_	Pezzoni et al. (2020)
rtRT-PCR TaqMan Probe	3D	-	Fluorescent intensity in real-time PCR detection system	10 TCID_50_	Pezzoni et al. (2020)
rtRT-PCR based on 2B-IR TaqMan Probe	5’ UTR	-	Fluorescent intensity in real-time PCR detection system	10 TCID_50_	Pezzoni et al. (2020)
rtRT-PCR based on 3-IR TaqMan Probe	5’ UTR	-	Fluorescent intensity in real-time PCR detection system	1 TCID_50_	Pezzoni et al. (2020)
RT-LAMP	3D	Incubation at 63°C within 30–60 min followed by an inactivation period of 2 min at 80°C	Agarose gel electrophoresis	approximately 50 viral RNA copies per assay	Blomström et al. (2008)

The Vesicular exanthema of swine virus (VESV, Caliciviridae, Vesivirus) can also cause vesicular disease in pigs. The origin of VES was traced to feeding meat from sea mammals to pigs and was eradicated from California populations in 1956. We also briefly refer to the laboratory diagnosis of VESV in this article.

## Diagnostic methods

### Reverse transcription-polymerase chain reaction (RT-PCR) assay

RT-PCR, one of the most traditional and common nucleic acid detection methods in the laboratory, is used extensively in various fields such as biology and medicine, microbiology and food based on its characteristics of simple operation, high sensitivity and strong specificity. Currently, multiple RT-PCR assays aimed at different viral targets have been reported and used as diagnostic tests.

FMDV is divided into seven serotypes based on genetic and antigenic analyzes. Meyer et al. designed primers based on the highly conserved regions of the seven FMDV serotypes genome and reported the establishment of a rapid and sensitive FMDV RT-PCR detection method (Meyer et al., 1991). Nishi et al. designed the primer set FM8/9 to amplify 644 bases in the conserved 3D region of all seven FMDV serotypes using RT-PCR assay, which is suitable for FMD diagnosis (Nishi et al., 2019). The study found that the sensitivities of FM8/9 primers were 10^0.6^- to 10^3.8^-fold higher than 1F/R primers described in the OIE manual (Nishi et al., 2019). There is a lack of cross-immune protection between different FMDV serotypes, and even within the same serotype, there are differences in the antigenicity of different isolates. Thus, it is necessary to develop a rapid detection method for distinguishing FMDV serotypes. FMDV VP1 coding sequences vary considerably among different FMDV serotypes, so that it can be used to RT-PCR assay for FMDV serotyping (Callens and De Clercq, 1997; Le et al., 2011). Even so, important phenotypic traits of FMDV cannot be reflected by relying on the VP1 coding sequence, since the FMDV genome is prone to variation and recombination events can occurr in the non-structural genes of FMDV (Carrillo et al., 2005; Klein et al., 2007; Brito et al., 2018). Xu et al. developed a universal L-P1 RT-PCR for amplifying and sequencing a 3 kb fragment including FMDV leader and capsid-coding region, which can be used for rapid antigenic characterization of FMDV and phylogenetic analyzes, and is expected to be an instructive for vaccine selection during FMDV outbreaks (Xu et al., 2013). A simple, quick and cost-efficient RT-PCR assay suitable for generating genomic sequences of all FMDV serotypes was described, and this approach can amplify the sequence reaching from the IRES to the end of the open reading frame, which can assist in immediate virus genotyping, phylogenetic analysis, and epidemiological studies of FMDV (Dill et al., 2017).

Since 2015, pig-producing countries such as Brazil (Leme et al., 2015), China (Wu Q. et al., 2017), the United States (Canning et al., 2016), Colombia (Sun et al., 2017), Canada (Xu et al., 2017), and Thailand (Saeng-Chuto et al., 2018) have successively reported cases of SVA-VD. SVA seems to be becoming a globally prevalent virus. Several RT-PCR assays based on SVA conserved genome have been established for viral diagnosis and epidemiological investigation of SVA (Laguardia-Nascimento et al., 2016). Leme et al. designed a primer set which can amplify a 542 bp product size of the VP3/VP1 region of SVA genome in RT-PCR assay, and the primer set can be useful in molecular screening of SVA infection and for characterization of the virus (Leme et al., 2015). Wu et al. designed RT-PCR primers for amplification of the whole SVA genome, and firstly carried out complete genome identification, phylogenetic analysis and clinical characterization of SVA that infected pigs with Porcine Idiopathic Vesicular Disease (PIVD) in China (Wu Q. et al., 2017). Transient viremia can be caused in SVA infected animals. In the meanwhile, viral nucleic can be detected in almost all tissues through RT-PCR. However, after viremia disappeared in affected animals, the virus can only be detected in the tonsils and lymph nodes using this method (Houston et al., 2020). A specific nested-PCR assay based on VP1 fragments of SVA genome was established and the limit of detection was 0.0181 ng/μL for the cell-cultured SVA isolate, which is more sensitive than traditional RT-PCR (Feronato et al., 2018).

Italy was the first country to report the outbreak of SVD in1966 and began an eradication program in 1995 (Nardelli et al., 1968; Bellini et al., 2007). So far, SVD monitoring is still ongoing in Italy and no evidence of SVD activity has been found, which indicated the complete SVD virus eradication from the Italian pig industry (Tamba et al., 2020). Whatever, SVDV has clinically similar symptoms with FMDV and affects trade activities such as pork products, as a result, it is necessary yet for diagnosis and surveillance of SVDV. Several RT-PCR assays based on different targets including capsid, VP1, 3BC and 2A genes were reported for SVDV detection and molecular evolution analysis (Marquardt and Ohlinger, 1995; Lin et al., 1997; Zhang et al., 1999). Nielsen et al. depicted a method for rapid sequencing of the SVDV isolates genomes using the Roche GS FLX Sequencing Platform (Nielsen et al., 2014). Vázquez-Calvo et al. designed PCR Primer sets and reported the first complete coding sequence of a Spanish SVDV isolate (SPA/1/'93)(Vazquez-Calvo et al., 2016). A double PCR method was described for detecting ASFV and SVDV (Peng et al., 2015). Pigs infected with SVDV can shed large amounts of the virus through the nose, mouth and feces to contaminate the surrounding environment. Given the absence of clinical signs, feces are the sample of choice for viral testing to identify subclinical infections (Pezzoni et al., 2020). Sensitive detection methods are necessary due to the low viral content of stool samples. The diagnostic performance of six genomic amplification methods for SVDV detection including RT-PCR, real-time RT-PCR and RT-LAMP assay was evaluated and the result showed that the conventional 3D RT-PCR and the 3D rtRT-PCR using SYBR Green as a detector were still the most sensitive and efficient methods for detecting SVDV in fecal samples (Pezzoni et al., 2020). However, compared with RT-qPCR, the procedure of RT-PCR is more complicated, and agarose gel electrophoresis is required to determine the results of the corresponding samples.

### Real-time PCR assay

As the gold standard of laboratory detection, the quantitative real-time reverse transcription PCR (qRT-PCR) assay has been successfully used in the diagnosis of various pathogenic pathogens (Munro et al., 2013; Shi et al., 2016; Fischer et al., 2017; Corman et al., 2020). qRT-PCR allows the quantification of molecular targets in templates by using standard substance. The sample content determination does not need to open the reaction tube, and can be analyzed by the fluorescence signal intensity generated during the qRT-PCR amplification process, avoiding cross-contamination between samples (Galluzzi et al., 2018). qRT-PCR has the advantages of rapidity and sensitivity, and is suitable for different clinical sample types including swabs, sera, vesicular fluid, milk and tissue samples (Burkhalter and Savage, 2017; Fontel et al., 2019; Yeo et al., 2020; Armson et al., 2020a). At present, Real-Time PCR is a commonly used method to detect the pathogens of animal diseases. Usually, the same diagnostic sample is repeated following a positive result to confirm the presence of the disease agent followed by secondary confirmatory testing if the disease is exotic. According to the OIE manual, qRT-PCR amplification of FMDV 3D genome sequence is recommended as a standard method for the viral diagnosis of all FMDV serotypes (Callahan et al., 2002). 5’UTR sequence is also the frequently-used target for FMDV detection (Reid et al., 2002). Both Reid et al. (2014) and Lim et al. (2022) established multiplex real-time RT-PCR based on FMDV VP1 coding region to detect and identify different FMDV serotypes (O, A and Asia). Normally, the FMDV RNA for diagnostic purposes can be harvested from infected animal samples containing blister fluid, blister tissue, serum, milk and swabs of lesion (Armson et al., 2019a; Wong et al., 2020). One study showed that EDTA-stabilized blood samples can be adapted to FMDV detection during FMD outbreak even though the sensitivity of it was 10-fold less than that of serum samples (Fontel et al., 2019). Armson et al. determined the utility of testing pooled milk by rRT-PCR as an alternative approach for FMD surveillance, and proved that pooled milk has potential value as a surveillance sample to reveal subclinical FMD infection (Armson et al., 2020a,b). Yeo et al. amplified the FMDV genomic sequence in meat juice using qRT-PCR and confirmed the presence of FMDV RNA (Yeo et al., 2020). The discovery indicated that meat juice can be used as a good sample type for FMDV detection and play a role in the import and export quarantine of meat products (Yeo et al., 2020). The application of qRT-PCR in low-and middle-income countries (LMICs) was limited because of the complex diagnostic procedures and expensive equipment (Vosloo et al., 2002; Namatovu et al., 2013). Recently, some of the filed-deployable molecular assays for FMD diagnosis were developed and proved the feasibility through research (Howson et al., 2017b, 2018; Hole and Nfon, 2019). Ambagala et al. described a potentially useful field-deployable reverse transcription-insulated isothermal PCR (RT-iiPCR) assay for rapid detection of FMDV in countries with poor equipment conditions (Ambagala et al., 2017). Moreover, a recent study described a portable, handheld real-time PCR platform that can be manipulated via a smartphone app, which has great potential for FMDV detection (Hole and Nfon, 2019).

qRT-PCR enables the fast and accurate diagnosis of any vesicular diseases including SVA, due to the characteristics of high efficiency and sensitivity (Leme et al., 2017). Different qRT-PCR targeting diverse SVA genomic conserved regions (including 3D, VP1 and 5’-UTR) were established for the SVA detection and quantification (Bracht et al., 2016; Gimenez-Lirola et al., 2016; Dall et al., 2017; Fowler et al., 2017; Zhang J. et al. 2019). Joshi et al. utilized real-time reverse transcriptase PCR and virus isolation to screen out the existence of SVA in pigs, mice and houseflies (Joshi et al., 2016). Several researchers detected the viral nucleic acid in tissue, serum, oral swabs and/or rectal swabs from pigs challenged with SVA by qRT-PCR, and found the low viral level in serum, which indicated that SVA can induce transient viremia (Fernandes et al., 2018; Buckley et al., 2021). Moreover, the study suggested that the tonsil may be the main site of SVA replication (Fernandes et al., 2018). Zhang et al. designed primers and probes based on conserved sequences of the viral genome, and established a SVV rRT-PCR targeting the conserved 5’-UTR and a SVV RT-iiPCR targeting the 3D gene, both of which have good consistency in the diagnosis process of clinical samples (Zhang J. et al., 2019).

A variety of real-time quantitative PCR methods have been described for the detection of SVDV (Reid et al., 2004a; Hakhverdyan et al., 2006; Niedbalski, 2009; McMenamy et al., 2011). The use of oral fluid (OF) samples to assess animal group health status has been proposed as an attractive and cost-effective method for disease surveillance, since this approach has no complex collection process and can be operated easily for anyone (Mur et al., 2013; Senthilkumaran et al., 2017). Senthilkumaran et al. utilized a qRT-PCR assay based on SVDV 3D target and an adapted competitive ELISA to detect the levels of viral nucleic acid and antibodies in OF samples (Senthilkumaran et al., 2017). The result showed that the high levels of SVDV RNA could be detected from 1 day post-infection (DPI) to 21 DPI and the antibodies to SVDV could be discovered starting at DPI 6, which indicated the potential value of OF for SVD surveillance (Senthilkumaran et al., 2017). Pezzoni et al. compared the sensitivity of the two real-time RT-PCRs targeted on the 3D region (based on SYBR Green and TaqMan probe detection), and they showed an equivalent analytical sensitivity, while the diagnostic sensitivity of the 3D rtRT-PCR using SYBR Green as a detector was high than the assay based on TaqMan probe detection (Pezzoni et al., 2020).

### Droplet digital PCR

**Figure 1:**
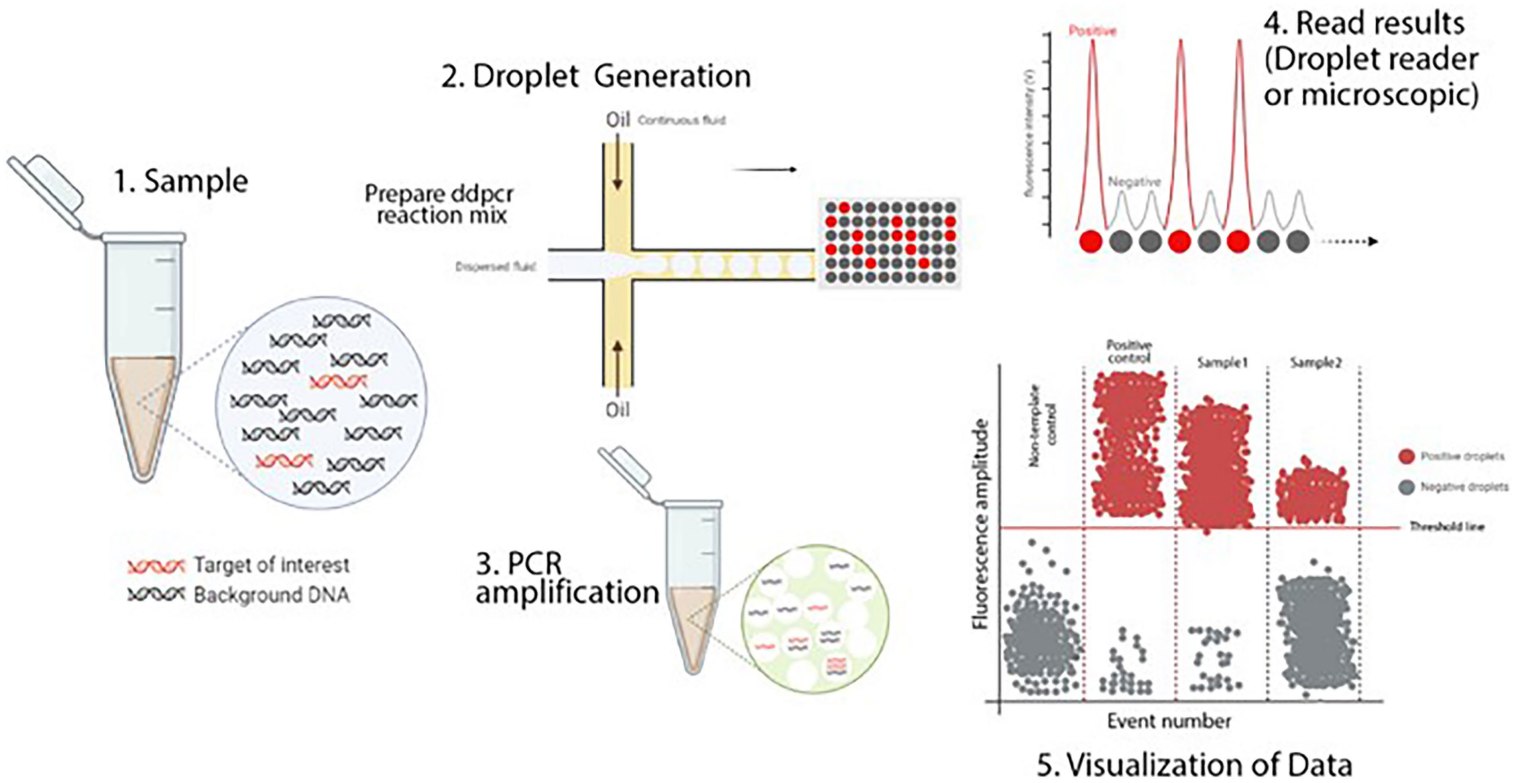
Schematic illustration of ddPCR. This figure illustrates the digital PCR droplet principle and how a single sample containing the target sequence is partitioned. PCR amplified produces thousands of copies that can be detected and interpreted by a detection system. In a typical ddPCR workflow, a single sample includes target and nonspecific sequences (DNA or RNA), real-time PCR primers and fluorescent-labeled probes, and standard real-time PCR master mixes. (1) A sample partitioned into thousands of single nanoliter droplets with the generation of water-in-oil emulsions. A proportion of droplets contain no template molecules, while others contain one or more targets. The generation of droplets from the sample is achieved in several ways. Standard methods include T-junction and flow-focusing geometry. Only the flow-focusing geometry is shown here. For more details, see the text. (2) Traditional end-point PCR is then performed to amplify the target sequence. (3) Target sequence droplets exhibit higher fluorescence intensity and are known to be positive droplets. Empty or no targets show low and negative fluorescent intensity. This fluorescence intensity versus time is plotted on a graph. The number of targets per partition will follow the normal Poisson distribution encapsulation of the DNA or RNA that occurs randomly. Various methods can interpret the fluorescent intensity of droplets. The most popular of these is a fluorescent microscope or a droplet reader. (4) The acquired data is visualized in a graph by different software. The threshold value indicates the intensity of fluorescence as the positive particles are separated from the negative. Although this value is set automatically by the software, it can be adjusted manually (Kojabad et al., 2021).

ddPCR is considered a graceful adaptation of the current qPCR format and has the potential application value on virus diagnosis (Jones et al., 2014). Its principle is shown in Figure 1. In ddPCR, the clinical sample is divided into thousands of droplets, each of which contains zero or several copies of the target nucleic acids. Molecular targets of each droplet are amplified by PCR and the fluorescence intensity produced by the reaction products is read after amplification, so as to achieve accurate and absolute quantification of target nucleic acid (Taylor et al., 2017). ddPCR does not depend on the standard calibration curve, and has a strong tolerance to PCR inhibitors so that it can realize the direct quantification of clinical samples including stool, blood, and tissue without nucleic acid purification (Taylor et al., 2017; Sahore et al., 2018). The rise of ddPCR is of great significance for the development of precision virus detection platform and viral diagnosis in the early period of infection. At present, dd-PCR has been widely used in the detection of various pathogens such as Epstein-Barr virus; human cytomegalovirus, human T-cell leukemia virus type 1, respiratory syncytial virus (RSV) and Japanese encephalitis virus (JEV) (Lin et al., 2016; Nicot et al., 2016; Wu X. et al., 2017; Persson et al., 2019). A study reported a RT-ddPCR method for the quantification of foot-and-mouth virus RNA, which is based on an OIE-recognized real-time RT-PCR that detects the 3D-encoding region of FMDV (Pinheiro-de-Oliveira et al., 2018). The assay can detect and quantify three serotypes of FMDV (O, A and C) with high specificity, and can detect at least 10^1.4^ TCID_50_/mL of FMDV and 2 fg of the circularized or linearized plasmid with the same analytical sensitivity of RT-qPCR (Pinheiro-de-Oliveira et al., 2018). Besides, T F Pinheiro-de-Oliveira et al. developed a one-step RT-ddPCR method with superior performance to identify SVA in biological samples (Pinheiro-de-Oliveira et al., 2019). Based on the high sensitivity and specificity, the one-step RT-ddPCR can be a useful tool for the prevention and control of vesicular diseases such as SVA (Pinheiro-de-Oliveira et al., 2019). Zhang et al. also established a novel RT-ddPCR assay targeting the conserved SVA polymerase 3D gene, and the detection limit of this assay was 10-fold more sensitive than the RT-rPCR assay (Zhang Z. et al. 2019). Currently, there is no digital PCR reported for SVDV.

ddPCR has the characteristic of accurate quantification and can quantify the mutated target genes by using a small amount of sample, which is important in the detection of rare mutation targets (Huggett and Whale, 2013; Milbury et al., 2014). In addition, it gives great benefits in fast and easy diagnosis for some diseases difficulted to identify accurately due to the high sensitivity and specificity of ddPCR (Li et al., 2018). However, ddPCR is still limited in use even in many modern diagnostic laboratories for routine testing (Liao and Huang, 2017). In terms of technology use, professional personnel is required for ddPCR operations and results reading. Anyhow, as a novel, sensitive, accurate and promising quantitative technique, ddPCR plays an important role in the monitoring of cancer, pathogens, water environment and so on (Li et al., 2018; Postel et al., 2018; Kojabad et al., 2021; Tiwari et al., 2022).

### Loop-mediated isothermal amplification

**Figure 2:**
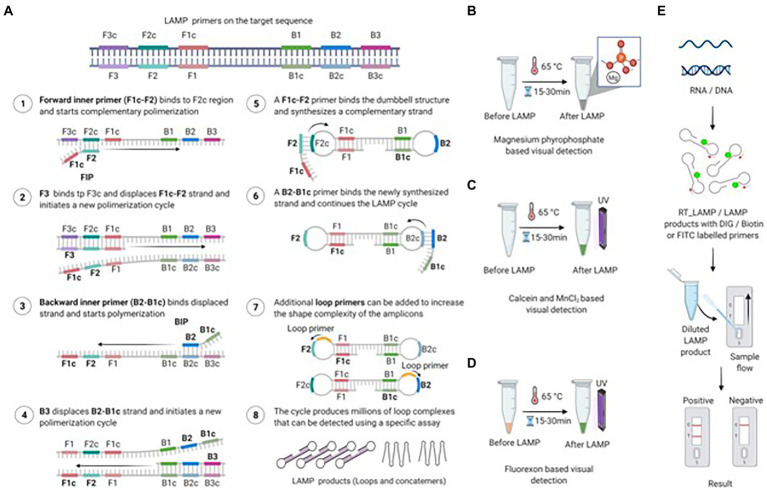
Amplification via loop-mediated isothermal amplification (LAMP) and various detection methods. **(A)** Diagram of LAMP primers located on the target sequence and the amplification process. The FIP primer binds to its complementary sequence and begins amplifying the first strand, followed by binding and amplification of the second strand by the F3 primer. The following steps involve the amplification of the FIP-amplified strand by the B3 primer. Finally, FIP and BIP continue the LAMP cycle, resulting in dumbbell-shaped amplicons. A full stepwise description of the individual steps is provided in the figure. **(B)** Magnesium pyrophosphate-based visual detection of the LAMP product. During LAMP, a large amount of pyrophosphate ion is produced as a by-product, which reacts with magnesium provided in the reaction mixture. The resulting product, magnesium pyrophosphate, forms a white precipitate that allows easy visual detection. The turbidity of the final LAMP reaction confirms the presence or absence of the targeted nucleic acid. **(C)** Calcein-based fluorescence detection of the LAMP product. The reaction of the by-product pyrophosphate with magnesium or calcein-manganese aids in the visualization of the LAMP product by producing a precipitate or emitting bright green fluorescence under UV light. Calcein-based fluorescence is enhanced by the presence of magnesium in the reaction mixture. **(D)** Fluorexon-based visual detection of the LAMP product. Upon completion of the LAMP reaction, fluorexon-MnCl turns from orange to green. The resulting green fluorescence is visualized with the naked eye under UV light. **(E)** Lateral flow-based detection of the LAMP product. To visualize LAMP with LFA, the target nucleic acid (RNA/DNA) is amplified via RT-LAMP or LAMP with DIG, biotin, or FITC-labeled primers. The labeled LAMP product is diluted and applied to the lateral flow strip. After 5 to 15 min of incubation, the appearance of bands on the test and control lines indicates the presence of the target nucleic acid (Bhat et al., 2022).

Loop-mediated isothermal amplification (LAMP) is an isothermal amplification approach developed in 2000, and has become the focus of point-of-care (POC) diagnostics due to its simple operation, rapid measurement and high sensitivity of this assay (Notomi et al., 2000). As shown in Figure 2 (Bhat et al., 2022), under the strand displacement activity of the Bst DNA polymerase from *Bacillus stearothermophilus* in 60-65^o^C, the target nucleotide was transformed into a stem-loop structure through the exploit of four pairs of primers (outward forward (F3), outward backward (B3), forward inner (FIP), and backward inner (BIP)). Then the stem-loop structure containing multiple initiation sites was used as the template of LAMP reaction for cyclic amplification, and many nucleotide chains with different lengths were generated finally. The results of LAMP assay can be visualized through the following ways (Nie, 2005; Almasi et al., 2014; Mansour et al., 2015; Peng et al., 2021; Bhat et al., 2022), (1) by agarose gel electrophoresis; (2) measuring the fluorescence emitted by SYBR Green or other dyes on a real-time basis; (3) the turbidity of LAMP reaction based on magnesium pyrophosphate; (4) the green fluorescence intensity based on calcein-manganese or fluorexon under UV light; (5) be visualized with a lateral flow device (LFD).

To date, several reverse transcription LAMP (RT-LAMP) assays have been developed that are capable of detecting all seven FMDV serotypes and serotype-specific detection of O, A, C and Asia 1 FMDVs (Lim et al., 2020). Timely diagnosis is essential for the control, monitoring and eradication of FMD. Compared with PCR, LAMP does not require complex temperature cycling conditions and requires less time to perform allowing it to be used for FMDV rapid detection in the field. The use of lyophilized reagents allows for pathogen detection in field environments where equipment conditions are insufficient. Howson et al. described the performance of lyophilized rRT-PCR and RT-LAMP assays to detect FMDV (Howson et al., 2017a). They found that lyophilization greatly improved the storage stability of test reagents without jeopardizing the assays' performance, which further supported the potential on field diagnosis of RT-LAMP assay (Howson et al., 2017a). Recently, Lim et al. developed a probe-based real-time reverse transcription loop-mediated isothermal amplification (RRT-LAMP) assay for rapid and specific detection of FMDV, and the detection limit of the assay was 10^2^ copies/μL which is comparable to that achieved by qRT-PCR (Lim et al., 2020). Rapid and accurate detection of SVA is necessary to confirm the disease causing agent, and to initiate the implementation of control processes. Zeng et al. described a real time RT-LAMP targeting the VP1 and VP2 regions of SVA and this assay can detect at least 1 TCID_50_/mL of virus titers (Zeng et al., 2018). Li et al. combined RT-LAMP with a lateral flow dipstick (LFD) for spot rapid diagnosis of SVA (Li et al., 2019). Armson et al. established SVV-1 RT-LAMP assay using lyophilized reagents, which negated the requirement for RNA extraction and the time of sample amplification was reduced within 12 min (Armson et al., 2019b). RT-LAMP technology is also used in the rapid detection of SVDV. Blomström et al. developed a one-step RT-LAMP assay based on the highly conserved 3D polymerase gene to test viral nucleic acid easily and quickly (Blomström et al., 2008).

Unlike PCR assays, LAMP does not require expensive equipment and complex cycling-temperature conditions, and can be easily performed by an untrained person. The designing principle of LAMP primers is a bit complicated. LAMP primers can be designed using the free online software package Primer Explorer version 5^1^ or paid software^2^ (Bhat et al., 2022). On the whole, LAMP is a potential tool for use of pathogen detection in resource-poor environments.

### Recombinase polymerase amplification

**Figure 3:**
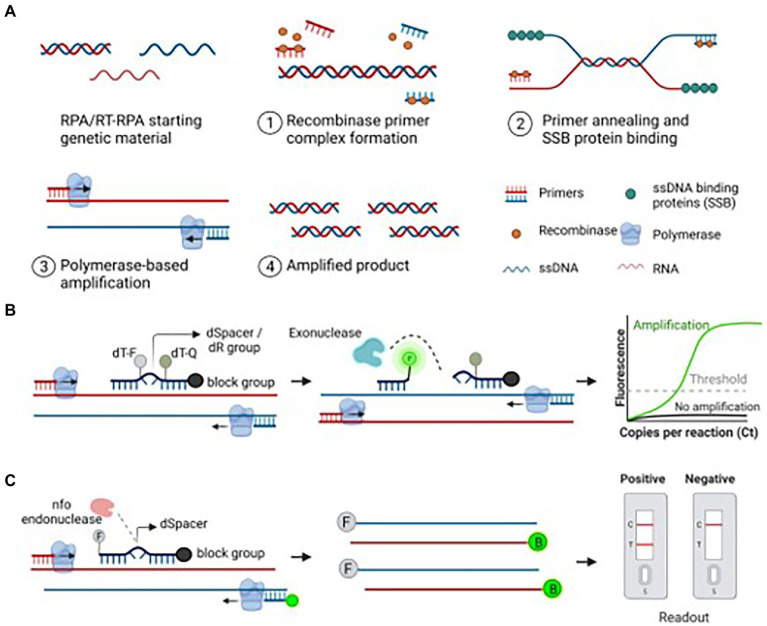
Recombinase polymerase amplification (RPA)-based amplification and its detection methods. **(A)** Schematic diagram of the RPA assay. An ideal RPA assay consists of forward and reverse primers, a recombinase protein that helps bind primers to the target nucleic acid, ssDNA-binding proteins to stabilize the ssDNA, and a polymerase to amplify the primer-bound strands. The stepwise amplification process is shown in the figure. **(B)** Exonuclease-based detection of the RPA product. A 46–52 bp long Exo probe flanked by a quencher and fluorophore binds to the amplified product. Exo probe contains a THF (tetrahydrofuran) residue known as dSpacer, which is cleaved by the exonuclease, thus releasing the fluorophore from the quencher. The observed fluorescent signal indicates the presence of the target nucleic acid. **(C)** Endonuclease-based detection of the RPA product. A 46–52 bp long oligonucleotide probe labeled with FAM or Alexa fluor binds to the target strand. The annealed probe is cleaved by the nfo enzyme, freeing the 3′-OH group of the probe and is used as a primer in the subsequent reactions. The resulting amplicons are produced with FAM and biotin using a biotin-labeled reverse primer. The final RPA product is applied to the LFA strip, and the results are visualized after the appearance of matching lanes (Bhat et al., 2022).

RPA was originally developed by Piepenburg and collaborators for the use of human pathogen detections, and was later applied in the diagnosis of different RNA targets (Piepenburg et al., 2006; Silva et al., 2018). Generally speaking, RPA reaction contains 3 main protein components: uvsX recombinase from T4 phage, DNA polymerase, and single-stranded DNA binding protein (SSB). As shown in Figure 3, the recombinase protein can bind with primers to form a complex that can recognize and pair complementary sequences of the template under constant temperature. Then the target nucleic acid is amplified in large quantities under the action of polymerase and binding enzyme. Recombinase-aided amplification (RAA) developed by Qitian (Wuxi, China) has similar amplification rules with RPA while the recombinant enzyme is obtained from *Escherichia coli* (Shen et al., 2019). Similarly, the results of RPA can be visualized by agarose gel electrophoresis while it is easy to appear smeared bands. To avoid this phenomenon, the RPA product can be purified to remove proteins and crowding agents prior to gel electrophoresis (Bhat et al., 2022). In addition, real-time fluorimeter and lateral flow assay can also be used to detect RPA products when using intercalating dyes and specific probes (Daher et al., 2016; Lobato and O'Sullivan, 2018; Powell et al., 2018). A RPA reaction is incubated at a single temperature of 25–42^o^C without the need for thermocycling, and the time of DNA amplification is usually between 5 and 30 min, which is faster than that of other nucleic acid amplification techniques such as PCR assay (Piepenburg et al., 2006; LaBarre et al., 2011).

RPA has been successfully used to detect several infectious agents, including viruses. Howson et al. defined the relative performance of RT-RPA and RT-LAMP for the detection of FMDV, benchmarked against rRT-PCR (Howson et al., 2017b). They found that RT-LAMP can detect FMDV RNA without RNA extraction, while accurate results by RT-RPA only obtained when using RNA extraction (Howson et al., 2017b). A visible and equipment-free RT-RPA combined with lateral flow strip (LFS RT-RPA) was developed to detect FMDV 3D gene (Liu et al., 2018). The FMDV LFS RT-RPA assay was performed successfully in a closed fist using body heat for 15 min, and the products were visible on the LFS inspected by the naked eyes within 2 min (Liu et al., 2018). Wang et al. established a series of serotype-specific RT-RPA assays combined with LFD to differentiate FMDV serotypes A, O or Asia 1, respectively, and the detection limits of these assays were 3 copies of plasmid DNA or 50 copies of viral RNA per reaction (Wang H. et al., 2018). Wang et al. developed a VP2-based real-time fluorescent reverse transcription RPA (rRT-RPA) assay to rapidly detect SVA, and the detection limit of this assay is 1.185 TCID_50_, which is comparable to that of a previously published rRT-PCR assay (Wang et al., 2021). A simple, rapid and accurate (RPA-LF) diagnostic assay for SVA detection was developed, and the optimal reaction conditions were incubated at 35 °C for 25 min, and the result was visualized directly on the dipstrip (Wang et al., 2022).

### Luminex (beads-based technology)

In 2001, Luminex technology, a bead-based detection platform, became the first multi-target detection technology approved by FDA for clinical diagnosis. Luminex has two core technologies including xMAP and xTAG. xMAP, a solution-phase array composed of spectrally distinct microspheres (“beads”), could detect up to 100 targets in a reaction by coupling probes of different targets with microspheres. The TAG sequence is a generic TAG unique to Luminex and consists of 24 bases. The primers with TAG sequence were mixed with nucleic acids for PCR amplification to obtain the target products containing TAG sequence. The TAG sequence specifically combined with the anti-TAG sequence on the beads to acquire a “beads-detector.” Then the fluorescence of each microsphere was detected and analyzed by flow cytometry. With the advantages of high throughput, rapidity, sensitivity and accuracy, Luminex technology has been widely used in many fields such as drug development, disease diagnosis and food safety. Wu et al. developed a PCR-based fluorescent Luminex assay for human papillomavirus (HPV) genotyping, and the assay may be used to provide critical clinical information for the early detection of HPV (Oh et al., 2007). A Luminex xTAG multiplex detection method with high sensitivity and specificity, was developed for the simultaneous detection of 11 porcine viral diarrhea pathogens (Shi et al., 2021). A total of 753 porcine stool specimens from five districts in Shanghai were detected using this assay and the surveillance confirmed that PEDV is still the main pathogen of porcine diarrhea (Shi et al., 2021). Wang et al. reported a multiplex Luminex assay for rapid detection and differentiation of the PRRSV field strains and vaccine strains (Wang et al., 2020b). Compared to RT-qPCR method, Luminex assay can achieve higher strain coverage since only two oligos may be required in the detection system (Wang et al., 2020b). A rapid, highly multiplexed nucleic acid assay employing Luminex™ liquid array technology was developed early for the differential diagnosis of FMDV, SVDV and other vesicular diseases pathogens (Lenhoff et al., 2008). Due to its high-throughput characteristics, Luminex technology enables multiplex detection of pathogens. A variety of commercial kits based on Luminex technology for different pathogens have also been developed, which provides convenience for the rapid diagnosis of different pathogens, especially those with similar symptoms (Krunic et al., 2011; Gray and Coupland, 2014; Glushakova et al., 2019).

### CRISPR-Cas technology

**Figure 4:**
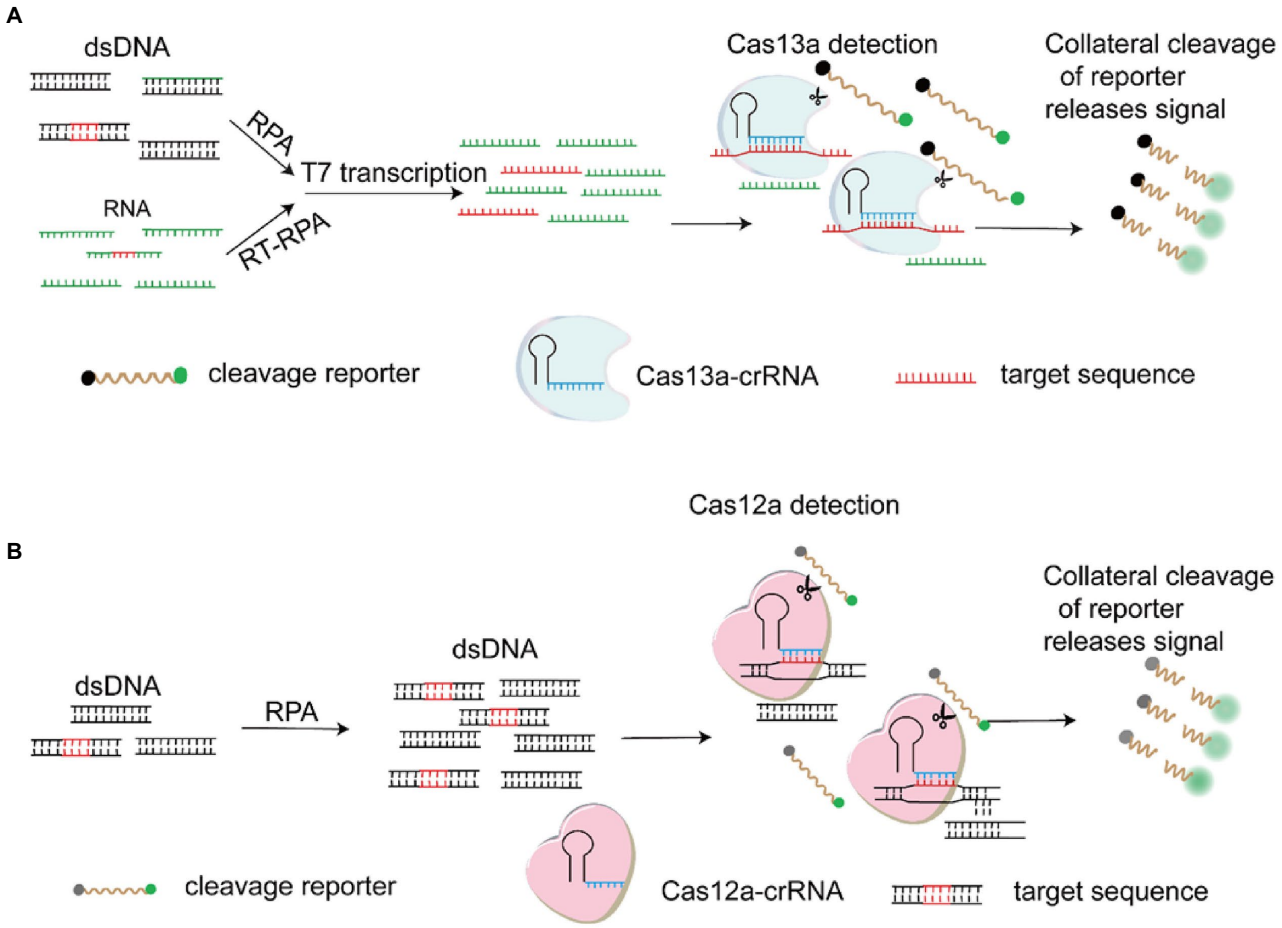
Schematic of the SHERLOCK and DETECTR. **(A)** CRISPR-Cas13a based detection (SHERLOCK). Following the RPA (Recombinase Polymerase Amplification) or RT (Reverse Transcription)-RPA, the RNA target recognized by Cas13a-crRNA complex is produced by in vitro transcription of the DNA amplicons. On recognition of the RNA target, the trans-cleavage activity of Cas13a is activated, resulting in the degradation of the doubly labeled fluorescent reporter. **(B)** CRISPR-Cas12a based detection (DETECTR). The RPA amplicon is used directly as the target for the Cas12a-crRNA complex. On the recognition of the DNA target, the trans-cleavage activity of Cas12a is activated, resulting in the degradation of the doubly labeled fluorescent reporter (Yin et al., 2021).

In order to deal with the threat of emerging and re-emerging infectious diseases, rapid detection of pathogen specific nucleic acid is essential. As a novel biosensing platform, CRISPR-Cas technology plays a key role in gene editing and molecular diagnosis. The system was originally discovered to resist the invasion of foreign DNA and viruses in bacteria and archaea (Wiedenheft et al., 2012). Subsequently, CRISPR-Cas9 system was developed as a powerful genome engineering tool and applied in various fields, especially in the treatment of genetic diseases, screening and detection of disease-related genes, cancer treatment, transformation of animals and plants, and prevention of pathogenic microorganisms (Yin et al., 2021). Cas12a is a single RNA-guided endonuclease lacking trans-activating crRNA (tracrRNA), which could recognize a T-rich PAM and exert the DNA cleavage activity (Zetsche et al., 2015). Unlike Cas12a, Cas13a targets and cleaves RNA at multiple locations without recognizing PAM (Yin et al., 2021). Intriguingly, both of the two nucleases exhibit collateral, nonspecific activities on random ssDNA or ssRNA respectively upon target recognition, so that they are respectively explored for nucleic acid detection (Yin et al., 2021). Diagnostic tool DETECTOR based on Cas12a (Chen et al., 2018) and SHERLOCK based on Cas13a (Gootenberg et al., 2017) were successively developed for DNA or RNA diagnosis (Figure 4). When the two nucleases recognized the corresponding targets, the trans-cleavage activity of Cas12a and Cas13a was activated to degrade the doubly labeled fluorescent reporter, which could produce fluorescence. CRISPR-Cas systems are developed as novel and powerful diagnostic techniques, and could use different strategies for virus detection including different signal readouts and lateral flow assays (LFA) (Yin et al., 2021). In general, good real-time PCR assays can detect 10 or less genome copies in a sample and the CRISPR-Cas assays can detect at similar levels. Meanwhile, combined with LFA, CRISPR-Cas technology is being developed as a sensitive, specific, rapid molecular diagnostic tool with potential, and shows great advantages in field-deployable detection. Multiple detection methods based on CRISPR-Cas12a/Cas13a were developed for different virus diagnosis such as influenza A viruses (Liu et al., 2019), SARS-CoV-2(Broughton et al., 2020), ASFV (Yang et al., 2022), PRRSV (Chang et al., 2020) and JEV (Xu et al., 2022). Multiplex detection of viruses

Different molecular biological detection methods including those mentioned earlier have played an important role in disease diagnosis and pathogen monitoring. PCR and other amplification reactions using different primer pairs simultaneously can quickly confirm the exact causative agent. Multiple specific primers pairs (including SVA, FMDV, SVDV and VSV) were used to identify SVA as the pathogen of vesicular disease in PIVD piglets from Guangdong and Hubei province, China (Qian et al., 2016; Zhang et al., 2018). However, these primers are reacted in separate tubes, so that it is time consuming, labor intensive and not cost-effective. The advantage of multiplex tests is that they increase the chance of identifying the microbial causes of viral infections and can detect more than one pathogen at a single time point when there are co-infections. Multiplex PCR (mPCR) is a validated strategy for the rapid detection and precise identification of a large number of viruses by incorporating several primers within one reaction tube to amplify genomic fragments of many pathogens. Fernandez et al. established a highly sensitive and specific one-step multiplex RT-PCR assay for the simultaneous and differential detection of FMDV, SVDV and vesicular stomatitis virus (VSV) (Fernandez et al., 2008). Lung et al. developed a multiplex RT-PCR and microarray for the detection/typing of 4 vesicular disease viruses (FMDV, VSV, SVDV, VESV) (Lung et al., 2011). In this study, multiple virus-specific probes for each of the four target viruses and serotype-specific probes for all VSV and FMDV serotypes were identified. Probe redundancy is an important advantage of microarrays over real-time RT-PCR in allowing broader coverage of diverse strains (Erickson et al., 2018). The use of a large number of probes increases the confidence of positive results and also decreases the likelihood of generating false negative results that may occur due to genetic changes resulting from virus evolution. Several other microarray assays have been described recently for the detection of vesicular disease viruses, but only two of these serotyped any viruses: FMDV (Baxi et al., 2006) and VSV (Baner et al., 2007). The assay used padlock probe methodology and both rolling circle amplification and PCR amplification. This assay targeted three vesicular disease viruses (FMDV, VSV and SVDV), but not VESV. Padlock probe technology has the capacity for highly multiplex assays and is promising for the detection and typing of genetically variable RNA viruses because the number of conserved regions on the target needed for amplification and detection is reduced when compared with conventional PCR amplification. The assay as described by Banér et al. had a run time that was comparable to real-time RT-PCR, but required separate addition of several reagents and the assay did not serotype FMDV. Furthermore, the sensitivity of the assay presented here appears to be higher for all three vesicular disease viruses targeted by Baner et al. (2007), although viral strains used for evaluation of the diagnostic sensitivity were different between the two studies. The single virus FMDV serotyping assay developed by Baxi et al. (2006) was expanded in this current work by including a multiplex RT-PCR for four vesicular disease viruses and a microarray that serotypes VSV in addition to FMDV. The additional information obtained regarding the presence or absence of differential pathogens may make the assay an attractive screening tool for vesicular viruses for use on a herd basis. Erickson and the colleagues combined PCR assay with microarray technology to develop a multiplex reverse transcription PCR and automated electronic microarray assay for detection and differentiation of FMDV, SVDV, VESV, CSFV, and ASFV (Erickson et al., 2018). Even though these assays with microarrays have been demonstrated to be effective, these microarrays are not used for diagnostics due to the cost and complexity of them. Shi et al. developed and validated a panel of multiplex real-time assays for simultaneous detection and differentiation of 12 important viruses and viral serotypes (Shi et al., 2016). The newly developed panel of multiplex real-time PCR assays offers a rapid, high-throughput, and reliable screening system for the 12 major viruses including FMDV and SVDV in swine (Shi et al., 2016). Recently, multiplex real-time RT-PCR assays for the detection and differentiation of FMDV and SVA was also developed and evaluated (Wang et al., 2020a).

## Concluding remarks

In recent years, with the growth of international trade, the risk of cross-border transmission of certain diseases has increased significantly. In order to prevent the spread of the vital pathogens that could harm economic animals, accurate diagnosis is essential. The possible disease agent can be determined through the observation of clinical symptoms and pathological changes of sick animals. However, the viruses with similar symptoms could not be distinguished such as FMDV, SVA and SVDV. Virus isolation is time-consuming and requires multiple blind passes of samples to produce usable amounts of infectious material. Further accurate and rapid confirmation of pathogens is required by serological and molecular biological methods. With the development of biotechnology, different molecular diagnostic techniques are constantly developing, which play a significant effect in the pathogen detection of swine vesicular diseases. RT-PCR and real-time RT-PCR are still the most reliable methods for detecting the viruses including FMDV, SVA and SVDV, among which real-time RT-PCR with high sensitivity is regarded as the gold standard. The development of multiplex detection provides a powerful technical means for the diagnosis of pathogens with similar clinical symptoms and the detection of co-infections. The combination of isothermal nucleic acid amplification method and lateral flow test can get rid of the limitation of instruments, save time and effort, and be helpful for rapid and accurate diagnosis on-site. How to improve sensitivity is the key to the development of this technology. Luminex technology has the advantages of high throughput, wide detection range and small sample size. In addition, it is suitable for large-scale screening of clinical samples, especially for the detection of multiple infections. The technology provides a new platform for high-throughput nucleic acid detection, and is widely used in the detection of various infectious diseases. CRISPR-Cas, characterized by sensitivity, specificity and programmability for nucleic acid recognition, has been repurposed for molecular diagnostics, thereby advancing a new path in biosensing. Disease diagnosis is an important part of epidemic prevention and control. New rapid and sensitive detection platforms applicable to the field are required to provide more time-saving and effective means in the outbreak of infectious diseases and minimize economic losses. Surveillance for FMDV and other viruses with similar symptoms including SVA, SVDV and VESV is necessary. The key to field diagnosis is the development of simple, low cost, user friendly, highly sensitive and specific rapid tests. This can be potentially accomplished using inexpensive, portable fluorescent instruments and test strips.

## Author contributions

WC: writing—original draft preparation. WW and XW: writing—review and editing. ZL, XL, and KW: conceptualization, supervision, and project administration. YL, LY, MZ, HD, SF, and JC: contributed to both the conception and design of the work. All authors have read and agreed to the published version of the manuscript.

## Funding

This work was supported by grants from the National Key R&D Program of China (2021YFD1800300); the Program of National Natural Science Foundation of China (No. 32172824); the Key Research Projects of Universities in Guangdong Province (No. 2019KZDXM026).

## Conflict of interest

The authors declare that the research was conducted in the absence of any commercial or financial relationships that could be construed as a potential conflict of interest.

## Publisher’s note

All claims expressed in this article are solely those of the authors and do not necessarily represent those of their affiliated organizations, or those of the publisher, the editors and the reviewers. Any product that may be evaluated in this article, or claim that may be made by its manufacturer, is not guaranteed or endorsed by the publisher.

## Footnotes

^1^ https://primerexplorer.jp/e/

^2^ www.optigene.co.uk/lamp-designer/
